# The Endoscopic Management of Different Pediatric Frontal Sinus Pathologies

**DOI:** 10.1155/2022/1078178

**Published:** 2022-02-10

**Authors:** Ali Almomen, Zainab Alshuhayb, Hussain Alsheef, Salma Alhammad, Balsam Alawami, Amirah Aldhurais, Hussain Almulla, Zahra Almoumen, Sarah Alkishi

**Affiliations:** ^1^King Fahad Specialist Hospital, Dammam, Saudi Arabia; ^2^Al Jaber ENT and Eye Hospital, Alahsa, Saudi Arabia; ^3^Maternity and Children Hospital, Dammam, Saudi Arabia; ^4^King Faisal University, Alahsa, Saudi Arabia

## Abstract

**Background:**

The paranasal sinuses in pediatrics can harbor a wide variety of pathologies. With the present literature being composed of case studies only, this entity is quite understudied.

**Objectives:**

This article aims to study the clinical presentation, diagnosis, and endoscopic management of six different rare frontal sinus pathologies in pediatrics, which include extensive allergic fungal sinusitis, mucoceles, osteoma, superior sagittal sinus thrombosis, CSF leak, and subdural empyema.

**Methods:**

We retrospectively studied all pediatric patients with frontal sinus pathologies presenting to our center, King Fahad Specialist Hospital, Dammam, Saudi Arabia, from the period of 2006 to 2020.

**Results:**

A total of 8 patients presented to our hospital with different frontal sinus pathologies. 5 of them were males, and 3 were females with an age of presentation ranging from 7 to 17 years. The diagnosis and localization were performed through computerized tomography without contrast and magnetic resonance imaging, when indicated. All cases were primarily managed with endonasal endoscopic approaches successfully without complications and with no recurrence evident upon follow-up.

**Conclusion:**

In this case series, six different frontal sinus pathologies were managed by an endoscopic approach, with excellent recovery and no recurrence upon follow-up demonstrated. This approach enabled excellent visualization of the pathologies, accurate localization, adequate drainage, and repair or grafting when needed.

## 1. Introduction

Although paranasal sinus disease has been extensively studied in literature, it has yet to be comprehensively studied in the pediatric population. The difficulty arises as a result of the variability in the size, shape, and pattern of development of each of the sinuses, along with a wide spectrum of pathologies that can affect them [1]. These pathologies include congenital malformations, traumas, neoplasms, and inflammatory etiologies [1].

Among all the paranasal sinuses, the frontal sinus is of particular significance due to its close anatomical relationship to the orbit and anterior skull base, which makes it vulnerable to complications, caused by either the disease or surgery [2].

### 1.1. Aim

The aim of this article is to showcase our experience and study the outcome of a set of six different frontal sinus pathologies in pediatrics. All were managed with an image-guided endoscopic surgical approach in King Fahad Specialist Hospital, a tertiary referral hospital in Dammam, Saudi Arabia.

## 2. Subjects and Methods

During the period from 2006 to 2020, all pediatric patients presenting to our Otorhinolaryngology Head and Neck Surgery department in King Fahad Specialist Hospital, Dammam with frontal sinus pathologies were enrolled in this study. Inclusion criteria included patients who were 18 years of age and younger at the time of presentation, with diagnosed frontal sinus disease, and managed solely by an endoscopic approach.

All patients were evaluated and diagnosed by CT scans and/or MRIs, when indicated, prior to surgical management. All patients were followed up postoperatively to assess resolution of symptoms and disease recurrence.

Ethical clearance was obtained from the Institutional Review Board (IRB) at King Fahad Specialist Hospital, Dammam. Written consent was obtained from all patients prior to the involvement of the study.

## 3. Results

### 3.1. Illustrative Cases

#### 3.1.1. Case 1: Pediatric Frontal Allergic Fungal Sinusitis with Intracranial Extradural Extension

A 15-year-old male presented to our ENT clinic complaining of nasal discharge, obstruction, and occasional headache. A detailed history of the present illness also revealed the presence of associated allergic symptoms. Past medical and surgical histories were unremarkable.

**Figure 1:**
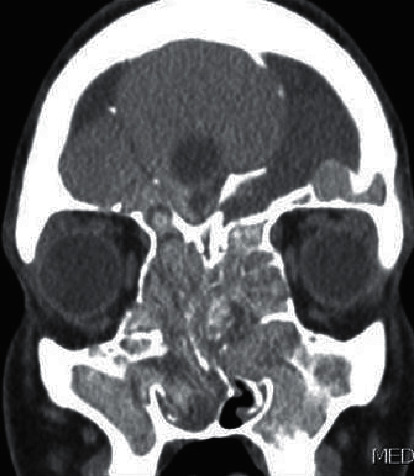
Computed tomography of the paranasal sinuses in the coronal plane, showing extensive disease with marked expansion of the sinuses, more pronounced in the right frontal sinus.

Noncontrast enhanced computed tomography of the paranasal sinuses revealed the presence of extensive disease with a marked expansion of the sinuses, more pronounced in the right frontal sinus, with dehiscence of the posterior wall and intracranial extension (Figure 1). Magnetic resonance imaging of the paranasal sinuses confirmed the right frontal sinus expansion and intracranial extension, limited by an intact dura, however.

For the management, the patient underwent navigated endoscopic sinus surgery, in which the following were performed: cleaning of the sinuses and the dehiscent pulsating dura from mucin with no CSF leak observed intraoperatively. After one year of following up with the patient, recurrent polyps have been noticed and managed with a short course of oral steroids. No recurrence has been noted after 3 years of follow-up.

#### 3.1.2. Case 2: Pediatric Lateral Frontal Mucocele

A 14-year-old female presented complaining of a one-month history of right frontal bone depression, associated with a headache. The patient denied the presence of nasal obstruction, discharge, and epistaxis. Detailed past medical and surgical history were unremarkable. Clinical examination in the form of nasal endoscopy and ophthalmic examination was unremarkable.

**Figure 2:**
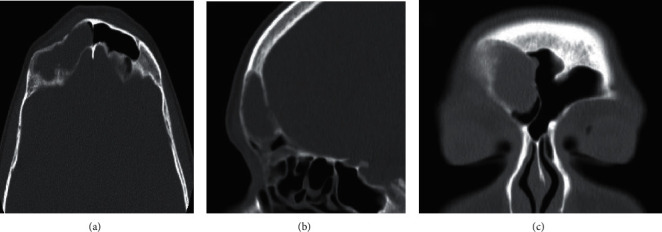
Computed tomography of paranasal sinuses in a bone algorithm in axial (a), sagittal (b), and coronal (c) planes. Right frontal sinus lesion with depressed fracture of the anterior wall and resorption of the lateral and posterior walls.

Noncontract enhanced computed tomography of the paranasal sinuses revealed the presence of the following: a low attenuation lesion filling the lateral aspect of the right frontal sinus, associated with thinning and resorption of the anterior, lateral, and posterior walls. The anterior wall showed posterior depression suggestive of a fracture (Figure 2). Magnetic resonance imaging of the paranasal sinuses excluded the presence of intracranial extension or thickening of the adjacent meninges.

**Figure 3:**
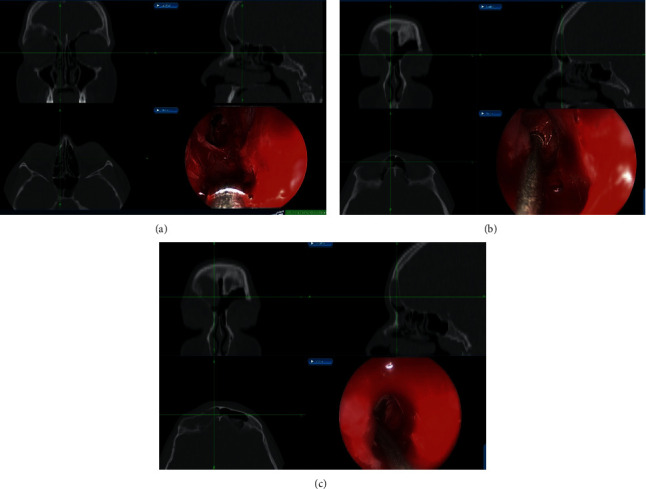
Endoscopic approach to frontal mucocele: Draf IIa wide frontal sinusotomy (a), 45-angled endoscopic view of the mucocele (b), and image-guided wide marsupialization of mucocele.

For the management, the patient underwent an image-guided endoscopic marsupialization of the right frontal mucocele, in which the following was performed: frontal sinusotomy (Figure 3(a)), mucocele identification by the aid of a 45-angled endoscope (Figure 3(b)), image-guided adequate marsupialization (Figure 3(c)), and removal of the anterior inferior thick wall. No complications or recurrence have been noticed after 2 years of follow-up.

#### 3.1.3. Case 3: Pediatric Frontoethmoid Osteoma

A 14-year-old male presented complaining of long-standing left-sided nasal obstruction, associated with a left-sided headache. Past histories of diagnosed sinusitis, allergic rhinitis, or epistaxis were denied by the patient. Clinical examination in the form of nasal endoscopy revealed the presence of a mass lateral to the middle turbinate on the left nasal cavity.

**Figure 4:**
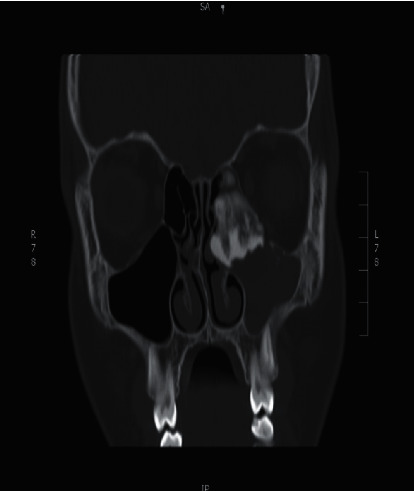
Computed tomography of paranasal sinuses in the coronal plane, showing a left ethmoid calcified soft tissue mass with inferior extension into the superior aspect of the left maxillary antrum.

Noncontrast enhanced computed tomography of the paranasal sinuses showed a left ethmoid calcified soft tissue mass with the following characteristics: an inferior extension into the superior aspect of the left maxillary antrum, heterogenous density with a ground glass appearance, calcified density at the periphery, and remodeling of the left medial orbital wall (Figure 4).

For the management, the patient underwent image-guided endoscopic sinus surgery to remove the lesion from the left ethmoid. The skull base and the orbital wall were preserved with no complications noted postoperatively. The patient demonstrated excellent recovery with no recurrence after 2 years of follow-up.

#### 3.1.4. Case 4: Pediatric Bilateral Frontal Mucopyoceles

A 17-year-old male presented to our ENT clinic complaining of long-standing facial heaviness, left-sided headache, bilateral nasal obstruction, anosmia, and recurrent episodes of epistaxis. A detailed past medical and surgical history revealed that the patient has diabetes mellitus type II, on insulin, and a surgical history of functional endoscopic sinus surgery, performed one year prior to presentation. Clinical examination revealed the presence of a displaced left eye downward and outward as well as bilateral nasal pale polyps grade 3-4 with white discharge.

**Figure 5:**
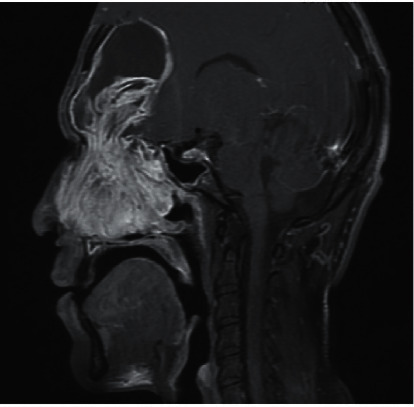
MRI of the brain and sinuses in the sagittal plane showing a significant expansion of both frontal sinuses caused by the mucopyoceles.

A noncontrast enhanced computed tomography of the brain and paranasal sinuses was performed preoperatively, in which the following was revealed: a marked enlargement of the frontal sinuses with the largest being on the left side, dehiscence of the posterior wall of the sinus, and intracranial extension of soft tissues within epidural space measuring 5.3 × 4.1 cm on the axial image. Magnetic resonance imaging of the brain and sinuses (Figure 5) confirmed the following: marked expansion of both frontal sinuses, more pronounced on the left side, measuring 4.7 × 6.5 × 6.7 cm with avid heterogenous enhancement, compressing and displacing the underlying dura with a marked mass effect.

**Figure 6:**
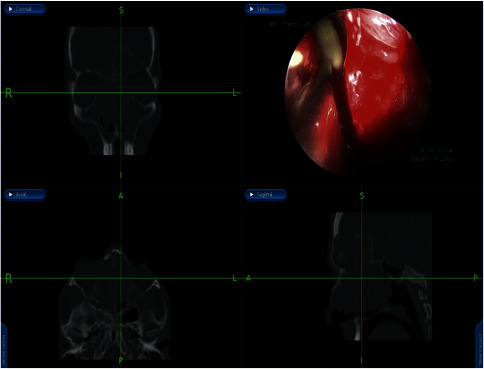
Identification of obstructing infected polyps in the frontal recess that were removed and drained by image-guided endoscopic sinus surgery.

**Figure 7:**
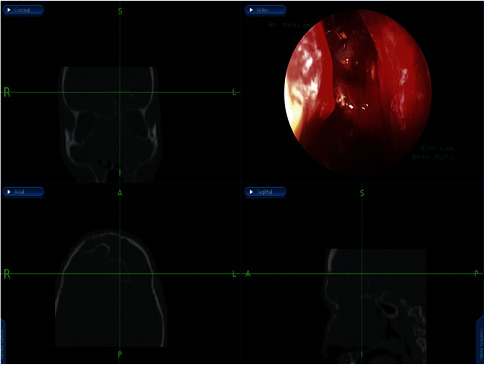
Draf type 2b performed, mucopyoceles cavities full of pus irrigated with antibiotic-soaked irrigations.

For the management, the patient underwent image-guided endoscopic sinus surgery, in which the following was performed: removal of the bilateral extensive nasal polyposis, grade 4, by a microdebrider with drainage of frank pus, removal of the obstructing polyps from the ethmoidal cavities, and removal and drainage of obstructing infected polyps in the frontal recess (Figure 6). Drainage and ventilation of the frontal sinuses were achieved by performing a Draf type 2b procedure bilaterally (Figure 7). The procedure facilitated the identification of the mucopyoceles cavities and the irrigation with antibiotic-soaked irrigation. Complete evacuation of both the mucopyoceles cavities was done and confirmed by the help of navigation. Complete disease resolution and normal aeration of the sinuses have been confirmed by the CT scan performed at the 6-month follow-up.

#### 3.1.5. Case 5: Pediatric Meningitis with Superior Sagittal Sinus Thrombosis

A 7-year-old female patient presented complaining of a two weeks' history of unresolving sinusitis with accompanying fever, headache, vomiting, and drowsiness. The patient was subsequently diagnosed as a case of bacterial meningitis secondary to acute frontal sinusitis.

The findings of the preoperative magnetic resonance imaging of the paranasal sinuses confirmed the presence of left frontal sinusitis, meningitis, and sagittal sinus thrombosis.

For the management, the patient underwent image-guided endoscopic drainage of the sinuses accompanied by a course of intravenous antibiotics administered postoperatively. An excellent recovery with no complications has been confirmed by the posttreatment MRI, performed upon follow-up.

#### 3.1.6. Case 6: Pediatric Subdural Empyema

An 11-year-old female presented to our clinic complaining of fever, headache, vomiting, and drowsiness. History taking revealed that the patient was diagnosed prior to presentation with acute sinusitis, unresolved with conservative management.

**Figure 8:**
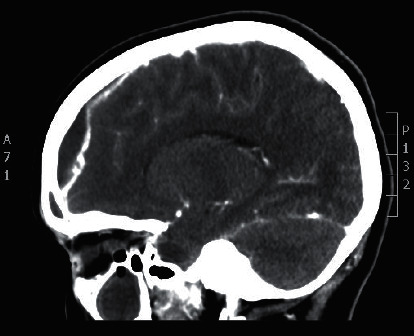
Sagittal brain CT scan showing a subdural empyema.

Noncontract enhanced computed tomography of the brain confirmed the diagnosis of a subdural empyema secondary to acute frontal sinusitis (Figure 8).

For the management, the patient underwent an image-guided endoscopic frontal sinusotomy and a frontal minicraniotomy, performed by the neurosurgery team, to drain the empyema. Surgical management was accompanied by a course of intravenous antibiotics. An excellent recovery with no complications has been confirmed by the posttreatment CT scan, performed upon follow-up.

#### 3.1.7. Case 7: Pediatric Frontal CSF Leak with Recurrent Bacterial Meningitis

A 10-year-old male referred to our ENT clinic with a history of two previous attacks of bacterial meningitis. Detailed history taking revealed a trauma to the head acquired while playing that preceded the attacks.

Noncontract enhanced computed tomography of the paranasal sinuses showed a suspicious frontal sinus defect. MRI and CT cisternography confirmed the site of the defect and excluded the presence of an associated meningocele.

**Figure 9:**
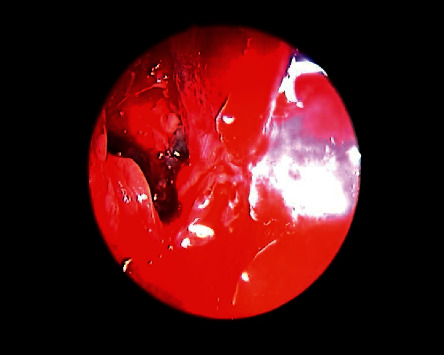
Endonasal endoscopic localization of the CSF leak in the posterior wall of the frontal sinus.

**Figure 10:**
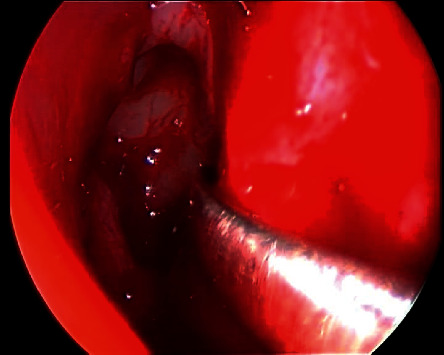
Endoscopic view showing the onlay free graft closure.

For the management, an endonasal endoscopic localization of the CSF leak situated in the posterior wall of the frontal sinus was performed (Figure 9) and managed by an onlay free graft closure (Figure 10). An excellent recovery with no complications has been confirmed upon follow-up.

#### 3.1.8. Case 8: Pediatric Traumatic Frontal CSF Leak with Meningocele

A 15-year-old male presented to our ENT clinic complaining of watery nasal discharge. A detailed history revealed that the patient was diagnosed with bacterial meningitis prior to presentation and had a trauma history of a recent motor vehicle accident.

**Figure 11:**
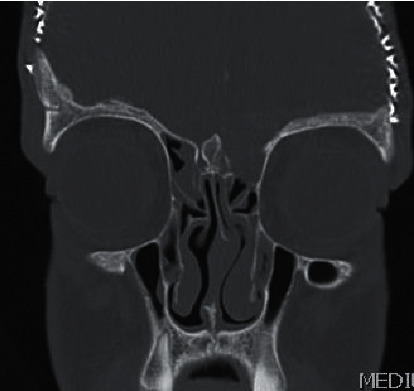
Computed tomography of the paranasal sinuses in the coronal plane, showing a frontal meningocele protruding through a frontal sinus defect.

Noncontract enhanced computed tomography of the sinuses revealed the presence of a frontal meningocele protruding through a frontal sinus defect in the coronal plane (Figure 11) and confirmed the presence of two frontal defects with the meningocele prolapsing through the posterior skull base defect in the sagittal plane. Endonasal endoscopic examination confirmed the diagnosis of the frontal defects with the meningocele.

**Figure 12:**
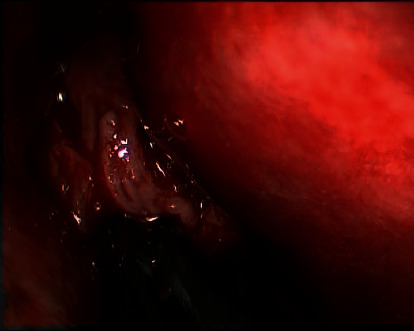
Endoscopic view showing two layers of free graft for skull base reconstruction.

For the management, two layers of free graft and a middle turbinate flap were placed for skull base reconstruction (Figure 12). The intraoperative image-guided navigation aided accurate localization of the defects and graft placements. An excellent recovery with no complications has been confirmed upon follow-up.

## 4. Discussion

Just like in adults, paranasal sinuses in pediatrics can harbor a large variety of pathologies. These pathologies range from self-limiting diseases to life-threatening conditions. In this case series, we present to you eight different cases of frontal sinus disease in pediatrics, which represent a fairly rare entity reported in the literature.

Acute sinusitis is highly prevalent in pediatrics [3–5]. The surgery comes into the scene when the infection causes intracranial or intraorbital complications. Among all the paranasal sinuses, the frontal sinus is the usual culprit when it comes to complications, with one study reporting rates of 11.4% if the frontal sinus was involved [6]. In a retrospective analysis of 16 pediatric patients who presented with intracranial complications from acute sinus infections, the most commonly involved sinuses were the ethmoid and the frontal sinuses [7]. Generally, intracranial complications are rare, with only 3% of pediatric patients developing such complications [8]. These complications include meningitis, subdural or epidural abscess, and cavernous sinus thrombosis [8, 9].

Diagnosis of acute sinusitis is especially challenging in pediatrics because patients often lack the typical nasal symptoms of the disease [10–12]; thus, most patients are diagnosed upon the development of complications. Intracranial complications should be suspected in any pediatric patient presenting with persistent high-grade fever, progressive headache, and neurological symptoms [13–15]. Two of these complications are demonstrated in our case series: superior sagittal sinus thrombosis and subdural empyema. Both of our patients typically presented with fever, persistent headache, and frequent emesis, similar to what is reported in other case reports [11, 16].

Subdural empyema is recognized in the literature as the most common sinogenic intracranial complication, making up to 85% of all complicated cases of sinusitis [17–23]. In a retrospective chart review that included twelve patients, subdural empyema was the most commonly reported intracranial complication [14].

Management of such cases generally includes the administration of broad spectrum antibiotics and surgical drainage [10, 16]. Both of our cases demonstrated excellent recovery upon the administration of antibiotics and endoscopic surgical drainage of the infected sinuses and empyema with no intraoperative or postoperative complications.

A mucocele is a mucous sac lined with an epithelial lining [24]. Frontal mucoceles are usually rarely encountered, with only sporadic reports existing in the literature [25–32]. The disease was first described in literature back in 1818 by Langenbeck with Rollet introducing the official term “mucocele” [33]. Identified underlying causes include chronic inflammation and trauma, with prior sinus surgery being the most common cause [33–35]. Mucoceles are managed surgically. The surgical approach may vary from endoscopic sinus surgery to craniotomy and craniofacial exposure, which ultimately depends on the extent of disease, whether intracranial or extracranial extension exists, and patient-specific characteristics [36]. Both of our cases were managed successfully with functional endoscopic surgery with no recurrence demonstrated upon follow-up, despite the presence of an intracranial extension in one of them. Other authors reported similar successful results. In one case series that included three pediatric patients with sphenoid and ethmoid sinus involvement, all cases were successfully managed with functional endoscopic sinus surgery alone [37]. The abovementioned cases, like ours, were ideal candidates for endoscopic surgery because of their somewhat limited extension and favorable anatomy. Endoscopic approaches are paramount when it comes to the pediatric population, as these approaches provide excellent results with no facial scarring and minimal morbidity.

Another pathology demonstrated in two of our cases is traumatic cerebrospinal fluid (CSF) leak. Underlying causes can be broadly divided into nontraumatic and traumatic, with the latter one being the most prevalent (80% of cases) [38, 39]. Trauma can be induced iatrogenically, most commonly following frontal craniotomy, and transsphenoidal hypophysectomy [40]. Both of our cases acquired direct trauma to the head prior to presentation. Depending on the severity of the case, the leak may be managed nonoperatively or operatively.

Frontal sinus cerebrospinal fluid leaks are rare, and their surgical management is difficult. Up until recently, they could only be treated by open surgery with an osteoplastic flap. With the development of endoscopic surgery, less invasive techniques such as an exclusive endoscopic approach are now used. Nevertheless, these techniques require a thorough knowledge of frontal sinus anatomy and endoscopic CSF leak repair. This knowledge is essential both to ensure closure of the CSF leak and to preserve frontal sinus patency [41].

In a case series of 10 pediatric patients with CSF leak, all cases were managed successfully using an endonasal approach [42]. Both of our cases were managed successfully using an endoscopic approach with no recurrence detected upon follow-up.

Paranasal sinus osteogenic tumors are an uncommon finding in pediatrics [43]. Out of these tumors, osteomas are the most common type reported, accounting for more than 66% of cases [43]. Proposed etiological factors include developmental, traumatic, and infective origins [44, 45]. There were no identified risk factors in our case. Conservative management is recommended when the osteoma is asymptomatic, slowly growing or occupying less than 50% of the frontal sinus [46, 47]. Otherwise, surgical excision is the rule. Since its addition in the early ‘99s, endoscopic surgical approaches have grown in popularity to manage such sinus lesions. In one published case series, a combined transnasal and transorbital approach was undertaken to remove a frontoethmoidal osteoma and was successful with no intraoperative or postoperative complications [48]. Our case was successfully managed with a transnasal endoscopic approach alone with no complications or recurrence upon follow-up. The choice of the approach was mainly based on the fact that the osteoma was confined to the ethmoid sinus with no extension beyond its border.

## 5. Conclusion

In this case series, six different frontal sinus pathologies were managed by an endoscopic approach, with excellent recovery and no recurrence upon follow-up demonstrated. This approach enabled excellent visualization of the pathologies, accurate localization, adequate drainage, and repair or grafting when needed. We recommend the use of this approach to be the rule, especially when the disease is confined to the frontal sinus, as it provides superior cosmetic outcomes along with minimal postoperative morbidity as opposed to open approaches.

## Data Availability

The data used to support the findings of this study are included within the article.
